# Commitment towards a better future for medical education in Saudi Arabia: the efforts of the college of medicine at Qassim University to become socially accountable

**DOI:** 10.1080/10872981.2019.1710328

**Published:** 2020-01-04

**Authors:** Saleh A Alrebish, Mohamed H. Taha, Mohamed H Ahmed, Mohamed Elhassan Abdalla

**Affiliations:** a Medical Education Department, College of Medicine, Qassim University, Qassim, Saudi Arabia; b College of Medicine and Medical Education Center, University of Sharjah, Sharjah, United Arab Emirates; c Department of Medicine and HIV Metabolic Clinic, Milton Keynes University Hospital NHS Foundation Trust, Milton Keynes, Buckinghamshire, UK

**Keywords:** Social accountability, social accountability grid, Qassim University

## Abstract

**Background**: The College of Medicine at Qassim University (COMQU) was founded in 2001 as a problem-based learning and community-oriented medical school in order to strengthen the health system not only in the Qassim region but also in the whole of Saudi Arabia. The aim of the current study was to assess whether the COMQU is a socially accountable medical school and the steps taken to achieve that target.

**Materials and methods**: The study used the social accountability grid published by the World Health Organization (WHO) as a framework to assess the social accountability efforts in the context of three functions of medical schools: education, service and research. Data were collected through the analysis of college documents (2001–2017) and interviews with key informants.

**Results**: The COMQU shows compliance towards social accountability in the three domains of the grid. The indicators related to the education domain demonstrate more compliance than those of research and community service in the grid.

**Conclusion**: The COMQU is based on community-oriented medical education (COME) with strong commitment towards social accountability (socially responsible going towards social responsiveness). More research is needed in order to pave the way to achieve social accountability status.

**Abbreviations**: COMQU: College of Medicine at Qassim University; COME: Community-oriented medical education; WHO: The World Health Organization; MOH: Ministry of Health; SCFHS: Saudi Commission of Health Specialties; PHC: Primary Health Care; HYMS: Hull York Medical School; WFME: World Federation for Medical Education; NCAAA: National Commission for Academic Accreditation and Assessment

## Introduction

The World Health Organization (WHO) defines the social accountability of medical schools as ‘the obligation to direct their education, research, and service activities towards addressing the priority health concerns of the community, region, and nation they have the mandate to serve’ [1]. It is worth mentioning that the social accountability of medical schools depends on the ways medical schools make a social contract with society (the way in which a medical school contributes to the welfare of society) [2] by producing graduates who will fulfil the health needs of the communities they intend to serve [3,4]. Ultimately the medical school-society relationship can be seen as a continuum.

The Global Consensus for Social The accountability of medical schools described in 2010 [5] defines three levels of this relationship. The first level is social accountability, which comprises four values – relevance, quality, cost-effectiveness and equity- in the context of the three functions of medical schools; Education, Research, and Community service [6]. The other two categories of the continuum levels are (a) social responsibility, which is the state of awareness the college has towards its duties to respond to society’s needs, and (b) social responsiveness, whereby the colleges has a course of action to address the current society’s needs [7].

These four values are the core of the grids of social accountability adopted by the WHO [8]

The College of Medicine at Qassim University (COMQU) was established in 2001 as a community-oriented and problem-based learning medical school. The COMQU was established in the Qassim region, central Saudi Arabia. The region has a population of 1,370,727 and an area of 58,046 km^2^ [9]. The educational programme lasts for six years and is divided into three phases. The first phase is the preparatory year. The second phase comprises the second, third and fourth years and covers the integrated body systems. The third phase is the clinical clerkship phase. Learning takes place in the community setting in primary health care centres in Phases II and III of the curriculum.

Early exposure to the community and clinical skills takes place early in the second phase, where students begin to identify the health care system and community needs. They also learn about basic communication and clinical skills, starting with role-play and ending with case histories taken from real patients.

This study aimed to assess the efforts of the COMQU to comply with the concepts of social accountability of medical schools using the WHO social accountability grid.

The WHO social accountability grid is a framework for measuring a medical college’s movement towards social accountability in each of the three domains of institutional responsibility: education, research and service [6].

## Materials and methods

This study is a qualitative case study. Data were collected through document analysis and interviews with key informants. Data were collected using the WHO framework for the evaluation of the social accountability of medical schools (Table 1). The document review included the following documents: the college programme/curriculum manual, student guide manual, the faculty members guide manual, reports prepared by the community service unit, student club reports, documents related to research carried out in collaboration with the Ministry of Health (MOH), publications from the college faculty members and students addressing the community issues in the Qassim region, and self-study reports that were prepared for accreditation. Most of data are available from the following link; https://med.qu.edu.sa/

**Table 1.** The WHO social accountability grid

	Domains and phases								
	Education			Research			Service		
Values	Planning	Doing	Impacting	Planning	Doing	Impacting	Planning	Doing	Impacting
Relevance									
Quality									
Cost-effectiveness									
Equity									

In addition, eight interviews with the college leaders, including the vice dean, head of the community medicine department, head of the community service unit, head of the research centre, three experts from the community and family medicine departments, and representatives from the directorate of MOH affairs in the Qassim region.

Interviews were conducted by two research assistants who have expertise in qualitative research and conducting interviews.

Interviews were semi-structured. Two of the authors carried out the interviews from February to April 2018, and each interview lasted between 60 and 90 minutes. A contact letter explaining the research was sent to the participants, in which they were assured of confidentiality and the right to withdraw from research at any time.

Following each interview, the informants were asked to nominate another participant who could take part in an interview to provide insightful information related to the study. Interviews were tape-recorded and transcribed verbatim.

Before reporting, the two authors plotted the contents of the obtained documents in the social accountability grid cells (Table 1) to generate a master sheet of data. Then, the plotted data were summarised, and a conclusion about the level of social accountability was reached using the categories suggested by Boelen [10].

Ethical approval was obtained from the Regional Research Ethics Committee of the Qassim region (Ethical Certificate No. 20180112, dated 12 January 2018).

## Results

The findings of this study are presented under the following headings.

The mission of the college states principles related to the education of graduates who are competent to respond the changing health needs of the society; the mission does not mention the principles of community service and research conduction as explicit functions of the college.

The mission statement is ‘Improving the health of the society by preparing competent health professionals who are able to respond to the changing health care needs and expectations of the community and are ready to excel in any field of medicine through high-quality student-centred and community-oriented medical education, by conducting applied research, by providing evidence-based health services, and by collaborating with national and international institutions’.

Our curriculum has well-defined a profile of the ideal doctor that would optimally respond to Saudi society’s health needs. Although we have encountered some challenges for assessing the relevance of this profile, as articulated in the mission statement, because health care delivery is a responsibility of the MOH, we should receive more collaboration from the MOH.

P 1

### Stakeholder involvement

The COMQU has a stakeholder council that is composed of a representative from each of the following groups: the MOH, General Directorate of Health Affairs, and Saudi Commission of Health Specialties (SCFHS), as well as a representative from the hospital directors and the community. The main goal of this council is to help the COMQU achieve its mission and to give regular feedback to the college leaders.

Furthermore, many of the college faculty are members of the regional health promotion committee, which is constituted by the MOH, e.g., in the regional health assembly at the Qassim region and the Forensic Medicine Committee at the Qassim region.

We believe that the college involved its stakeholders in curriculum implementation to a high degree through their involvement in the college’s advisory board, evaluation of their satisfaction with its graduates through the employment satisfaction survey, and the regular meetings conducted during the curriculum update process.

P 2

The COMQU has developed tools that ensure community involvement in the programme by examining the stakeholders’ extent of satisfaction with the programme and graduates. Three main extensive studies took place in 2012, 2016 and 2018. The university and the college also have an in-place employee survey that regularly assesses the extent of the employees’ satisfaction with the programme, graduates and the services provided.

### Education domain

The present study also revealed that 12 credit hours (7%) of 180 credit hours of the curriculum are devoted to learning about community issues, and learning takes place in the community setting in primary health care centres in Phases II and III. Early exposure to the community takes place early in the second phase, where students begin to identify the health care system and community needs. Moreover, there is a six-week course in family medicine at the end of the programme, in which the students consolidate their learning in the community setting.

The learning sites for our students are carefully selected to enable them to develop the necessary knowledge, skills and attitudes to successfully practice high-quality health care. We believe our students are active participants: they see patients, interact with teams of health professionals, and understand the administrative and policy issues that affect the health care delivery. Still, we need to increase the exposure of our students to community-based learning settings.

P3

No community placement of students takes place during their clinical training in the existing curriculum. The COMQU is currently reviewing the curriculum to align it with the national qualification framework and the Saudi Medical Education Directives, the SaudiMEDs Competency Framework, which is expected to improve the fitting to the Saudi community. Table 2 shows the plotting of the education-related to education domain on the social accountability grid.

**Table 2.** Application of the social accountability grid to activities of the educational programme, research, and community service of the college of Medicine, Qassim University, Saudi Arabia

	Domains and Phases								
	Education			Research			Service		
Values	Planning	Doing	Impacting	Planning	Doing	Impacting	Planning	Doing	Impacting
Relevance	The community needs to appear in the mission; the COMQU should adopt SaudiMEDs Framework & National Qualification Framework.	Medical students are introduced to the concepts that address the priority of health care issues.	The COMQU advisory board sets up a partnership between education professionals, policymakers and the community.	Research priorities are determined by the deanship of research at the university level.	Qassim University encourages researchers by funding research priorities.	Research outcome is documented by the research centre; the COMQU has KPIs.	Through the college advisory board, the QUCOM seeks to anticipate what types of services will be needed in the future to improve the health care and health status of the community, region or nation.	Faculty and students provide health service to thecommunity.	Community service unit activities, medical caravans and health education.
Quality	The COMQU updated the curriculum on a regular basis.	Learning sites are carefully selected and evaluated using students and graduate surveys.	Suggested changes from the stakeholders are implemented accordingly; new objectives and curriculum contents are added.	Research proposals are reviewed and approved by the college IRB and the regional IRB.	Qassim University encourages applied and operational research.	Research done by the college staff has influenced policy change.	The COMQU has an employee survey	Needs to be addressed	Needs to be addressed
Cost-Effectiveness	Needs to be addressed	Needs to be addressed	Needs to be addressed	Needs to be addressed	Needs to be addressed	Results of research in the priority areas are being published and presented in conferences in the college (e.g., on research days).	Needs to be addressed	Needs to be addressed	Needs to be addressed
Equity	The curriculum is designed to serve all community sectors; 20% of training is community-based.	Students are trained in all community settings, including underserved communities.	Needs to be addressed	There is no clear policy about conducting research in underserved communities.	Needs to be addressed	Needs to be addressed	Needs to be addressed	Needs to be addressed	Needs to be addressed

### Research domain

The research in the COMQU is overseen by the research centre, which regulates the process of research proposal writing for grants with the deanship of research at the university level and ensures the existence of the technical and ethical components of the research proposal before submission to the Regional Research Ethics Committee in the Qassim region. Despite the existence of the research centre in the college, the respondents stated that the research priorities of the COMQU were not identified in consultation with health care beneficiaries, health care providers and health care policy decision-makers. However, since 2011, the faculty of the COMQU has had many research projects at different areas of the health system, notably health care delivery, primary care, health promotion, disease prevention and health care teams. The interviewees also reported that the impact of research on the community had not been measured ever since the COMQU establishment.

Many staff members had conducted several kinds of research addressing the local community needs, and some of these studies were conducted jointly with the staff of MOH, However, these studies were conducted according to researchers interest. It would be useful if the research priority were determined in advance with input from a committee aware of the community needs.

P4

Several types of research have been conducted by the staff of the COMQU, which address the community local health system needs; some of them have been jointly conducted by the COMQU and MOH staff (Table 3). To encourage research and scholar productivity, the college is publishing a medical journal; the informants consider it to be a source of motivation to conduct research.

**Table 3.** Assessing the COMQU programme based on the social obligation scale

Elements of the obligation scale	Responsibility	Responsiveness	Accountability
Social needs identified	**Implicit**	**Explicit**	Anticipative
Institutional objectives	**Defined by faculty**	**Inspired by data**	Defined by society
Educational programme	**Community-oriented**	Community-based	Community-based
Quality of the graduates	**Good practitioners**	**Meeting criteria of professionalism**	Health system change agents
Focus of evaluation	Process	**Outcome**	Impact
Health partners	**Internal**	**External**	

The data showed that there is no policy concerning research on underserved communities. The college research unit had started developing a plan that was directed to the community priority health needs in accordance with the MOH plan. This unit works closely with the deanship of research at the university level. Some of the faculty studies are fully sponsored and funded by the university and are dedicated to the fulfilment of the set criteria.

The university administrates support for conducting studies in the priority areas with at least as much equipment, supplies, and funding as necessary. This support is reflected by the number of studies that has been conducted in the last few years

P 5

In the college, research is part of faculty members’ duties and not only for promotion. However, the impact of this research on the community is not mentioned in the rules to assess research productivity. Moreover, the faculty members are involved in educational research activities through acting as supervisors, subject matter experts and members of the thesis defence committee for students’ annual research projects as well as the electives and graduation mini-projects. Students’ studies, supported by the community medicine department, are directed towards community needs and problems.

### Service domain

The college established a community service unit in 2012, which aimed at providing health education in communicable and non-communicable diseases and medical caravans to the community. The community services unit provides many activities that take place in the community. The mandate of the community service unit emphasises ‘the promotion of health services provided to the community through participation of faculties in the provision of activities in the areas of diagnosis of community health problems, health education, medical training, and treatment, and partnership with community organisations with periodic feedback from the stakeholders in order to target the needs of society’. This study shows that all the community service originated from the staff of the COMQU and is not explicitly mentioned in the college mission.

The community service unit had a remarkable role in addressing the community needs of the Qassim region. Moreover, the university is developing a new university hospital, which will add to the health services provided to the community. This domain needs further collaboration with the directorate of health affairs at the Qassim region

P 6

Both faculty members and students provided health services to the community through the university clinics and primary health care (PHC) centres.

Table 2 shows the application of the WHO social accountability grid to the activities and outcomes of the educational programme of the COMQU – service domain.

### Assessing the COMQU according to the social obligation scale

We finally assessed the COMQU using social obligation scale as a way to consider the extent of a medical school’s social obligation about the particularities of the concepts of social responsibility, social responsiveness and social accountability. These are then positioned against six elements, with social accountability being regarded as the most desirable level of social obligation. The analysis of the existing documents and the interview transcripts show that the COMQU could be considered a socially responsive college of medicine (i.e., between social responsibility and social accountability) (Table 3).

## Discussion

For a school to be socially accountable, it needs to exhibit consistency between the stated goals and the programme implementation, outcome and impact [11]. In the case of a socially accountable school, the focus of evaluation is on the impact on people’s health status, which implies that the school is eager to follow up on how its products are ultimately used [5,12].

This study showed that the COMQU has had early exposure to the community training, which took place early in the second phase of the curriculum. This finding is in line with other findings in the Hull York Medical School (HYMS) in the UK, which is categorised as a socially accountable school [1]. There is early exposure to the community at the HYMS; however, this exposure takes place through clinical placements in primary health care clinics, which helps students contextualise their learning. In the COMQU, the component of community-based learning and health concerns in the community has individual courses worth 12 credit hours, and it is also integrated into all the courses of the college, representing approximately 10% of the weight of the contact hours of the whole curriculum. This was found to be short compared to the HYMS, which has fully integrated primary and secondary care placements, with an overall of 33% of time spent in the community [1], including longitudinal exposure to community tutors within practice placements (16-week placements in Years 3 and 4; eight-week apprenticeships in Year 5). Similarly, it was noticed that at the COMQU, students’ contact with community-based learning and community placement was very limited, compared to 25% in the Faculty of Medicine at the University of Gezira in Sudan [13].

There is also some evidence that having most of the undergraduate training in the community or community health units rather than in colleges and teaching hospitals would improve graduates’ skills and competencies [14].

Our study also found that the COMQU is undergoing curriculum reform according to the national qualification competency-based framework (i.e., Saudi Medical Education Directives, the SaudiMEDs Competency Framework). By doing so, the COMQU is trying to achieve the objective of the thematic Area 4 in the thematic areas of global consensus included in the social accountability requirement of medical schools published in 2010 (Table 1) [15]. The COMQU also obtained recognition from the World Federation for Medical Education (WFME) in 2013, and recently, it was accredited by the National Commission for Academic Accreditation and Assessment (NCAAA), the school accreditation facility in Saudi Arabia, which satisfies the requirement of Area 8 of the global consensus framework. It was noticed that, despite the Declaration of Tokyo three decades ago, the WHO expressed concern about the failure to modify the accreditation criteria of medical schools to better reflect people’s priority health needs. There were recommendations to incorporate social accountability into the standards of accreditation; we could not find any areas in the national standards in the KSA (i.e., the National Commission for Academic Accreditation and Assessment NCAAA) that incorporate social accountability [16].

If social accountability becomes a criterion for the evaluation and assessment of medical colleges, this will encourage the colleges of medicine to be more socially accountable [17]. Regarding setting research priorities and conducting research that addresses community needs, the respondents commented that the research priorities of the COMQU were not identified by health care beneficiaries, health care providers and health care policy decision-makers. However, the faculty of the COMQU has conducted many research projects at different levels of the health system, especially in areas such as health care delivery, primary care, risk assessment, health promotion, disease prevention and health care teams. The impact of research on the community has not been measured yet, which could be attributed to the fact that many sectors are involved in evaluating this impact (e.g., MOH).

Concerning cost-effectiveness and equity, as two values in the service domain of the WHO social accountability grid, this study found that the COMQU will need to do more work to improve cost-effectiveness and equity in the service and research domains. We believe that after the inaugural period of the university hospital, these two areas would be improved. This study showed that the COMQU has a unit responsible for providing services to the community, known as the community service unit (the need to define more key activities in order to increase community representation). This unit could form the basis for the college to build a proper partnership with the community and health system. Studies also suggest that establishing formal partnerships between local communities, health services and the health professionals contributes significantly to quality teaching, research and advocacy activities, which are relevant to local health services and population needs [18]. This study revealed that the COMQU could be best described as socially responsive – a midway point between socially responsible and socially accountable on the social obligation scale (Table 3). Social responsiveness can be defined as a course of action taken by the college to address society’s needs [1].

The medical schools are divided into three categories based on the extent to which they address community health needs: (a) a neutral medical school: in this category, a medical school performs its functions with little concern for adapting them to the changing needs of individuals, families and the community at large; (b) a reactive medical school: in this category, a medical school is aware of the health needs of society and reacts accordingly and responsibly; and (c) a proactive medical school: in this category, a medical school continually anticipates new developments and takes steps to address them [19]. In a proactive medical school, the school uses its resources to define future challenges in the health system and works with partners to invent and implement plans to address the expected challenge [20].

This study is not without limitations. One of the limitations of this study is that it relies on the analysis of the existing documents and the views of the college leaders and staff members. Further studies are needed to evaluate the COMQU’s interventions in the community (e.g., THEnet evaluation framework), in addition to involving undergraduate and postgraduate students [8]. Despite the COMQU’s mission of improving the health status of society by preparing competent health professionals who can respond to the changing health care needs and expectations of the community, our findings revealed that the impact on community health has not yet been measured. This may be due to the COMQU’s dependence on its stakeholders in this regard, namely, the directorate of health affairs at the Qassim region, as he/she controls data on the community health. Our findings suggest that a segment of the health workforce may need to be trained. Despite these limitations, our findings are novel, and this is first study about the social accountability of a medical school in Saudi Arabia. This novelty also explain why it was impossible to make comparisons with other medical schools in Saudi Arabia.

## Conclusion

Applying the WHO social accountability grid (Table 1) illustrated that the COMQU is achieving developments related to social accountability (Tables 2 and 3). However, the college needs to have more interaction with stakeholders to move away from the traditional COME path to the full spectrum of social accountability. The college needs to pay particular attention to the following areas: (a) health issues in the region and not only the health system/care issues, (b) issues related to the social determinant of health, and (c) cost-effectiveness in both education and research.

Further studies are required involving the students, graduates, and the other related stakeholders, and multi-centre studies should be conducted.

## Data Availability

The datasets used and/or analysed during the current study are available from the corresponding author on reasonable request.
